# Design and Optimization of Thin-Walled Main Support Structure for Space Camera Based on Additive Manufacturing

**DOI:** 10.3390/mi15020211

**Published:** 2024-01-30

**Authors:** Jiahao Peng, Shijie Liu, Dong Wang, Anpeng Xu, Xin Huang, Tianqi Ma, Jing Wang, Hang Li

**Affiliations:** 1 Changchun Institute of Optics, Fine Mechanics and Physics, Chinese Academy of Sciences, Changchun 130033, China; hellopjh@yeah.net (J.P.); xap_123@163.com (A.X.); huangxin19@mails.ucas.ac.cn (X.H.); jlspmatq@163.com (T.M.); wangjing@ciomp.ac.cn (J.W.); lixing20@mails.jlu.edu.cn (H.L.); 2 University of Chinese Academy of Sciences, Beijing 100049, China; 3 China North Vehicle Research Institute, Beijing 100072, China; wangdong173@mails.ucas.ac.cn

**Keywords:** wide-field auroral imager, main support structure, integrated thin-walled structure, active fitting optimization algorithm, additive manufacturing

## Abstract

In order to solve the design requirements of high stiffness and lightweight for the primary support structure of a wide-field auroral imager, we propose a solution for designing and optimizing a large-scale complex thin-walled structure using additive manufacturing. Firstly, we devise an integrated thin-walled structure and test material for the main support. Secondly, shape optimization is achieved via the optimization of the lateral slope angle of the primary support based on Timoshenko cantilever beam theory. Additionally, an active fitting optimization algorithm is proposed for the purpose of refining the wall thickness of the thin-walled structure. Then, we determine the structural design of the main support. This primary support is manufactured via selective laser melting (SLM). Following processing, the structure size is 538 mm × 400 mm × 384 mm, and the mass is 7.78 kg. Finally, frequency scanning experiments indicate that, in the horizontal direction, there is a natural frequency of 105.97 Hz with an error rate of approximately 3% compared to finite element analysis results. This research confirms that our large-scale complex, thin-walled main support structure design meets all design requirements.

## 1. Introduction

Observational research and assurance services for the space environment can help to reduce the risks posed by extreme space weather to aerospace, navigation, communications, and national economies [1,2,3]. Fengyun meteorological satellites have been extensively utilized in disaster prevention and mitigation, addressing climate change, and monitoring and predicting space weather. This use has yielded significant achievements [4,5,6]. The wide-field auroral imager (WFAI), a principal payload on Fengyun-3 polar-orbiting meteorological satellites, is specifically designed to capture data during periods of auroral radiation intensity within the 140 nm to 180 nm wavelength range. These observational data enable the provision of forecasts for magnetic storms, magnetospheric substorms, as well as ionospheric conditions within polar regions [7].

Considering the FY-3(08) satellite (WFAI), it can be observed that the WFAI employs the swing-sweep method within the −90° to +90° range to achieve a dual-channel 130° × 130° expansive field of view, as well as to enable on-orbit calibration. The WFAI is strategically positioned in the central region of the satellite’s payload bay. In order to mitigate any interference arising from other payloads on the satellite, the main support structure’s dimensions, oriented perpendicularly to the satellite payload bay plate, must be adequately extensive. Furthermore, the primary optomechanical structure of the imager is situated atop the main support structure, resulting in the camera displaying an elevated center of gravity. Simultaneously, the dimensions and mass of the WFAI in other orientations are subject to stringent constraints, and they must adhere to safety margin specifications during launch conditions. Consequently, these issues present challenges in crafting a main support structure for the camera, which is simultaneously lightweight yet characterized by high stiffness and stability.

This study predominantly focuses on the application of laser additive manufacturing to create large-scale, complex, thin-walled main support structures that can serve as space cameras. Initially, operating according to the design requirements of the wide-field auroral imager, namely, lightweight and high stiffness, an integrated thin-walled structure is devised for the main support. Subsequently, the lateral slope angle of the main support is optimized in accordance with the Timoshenko cantilever beam theory. Furthermore, an active fitting optimization algorithm is proposed for the refinement of the thin-walled structure’s thickness. Finally, considering the limitations of traditional methods in processing, selective laser melting (SLM) is employed to fabricate the main support. The vibration natural frequency test validates the optimal design results presented in this paper. The remaining sections of this research paper are organized as follows. In Section 2, we review the related literature. In Section 3, we summarize the design requirements and structural form of the camera. In Section 4, the original design of the main support structure is accomplished. In Section 5, the optimization-based design of the main support structure is performed, including shape optimization and size optimization. In Section 6, we introduce the additive manufacturing process of the main support structure and conduct experimental verification of the space camera. Conclusions and future research directions are given in Section 7.

## 2. Literature Review

Thin-walled structures play crucial roles in the aerospace field due to their performance advantages, such as being lightweight and exhibiting high specific stiffness, specific strength, and high-load-bearing capacity [8,9,10]. Possessing excellent temperature control characteristics and strong energy absorption performance, thin-walled structures are widely utilized in the main support structure of space cameras. The principal forms of thin-walled main support structures produced for space cameras include the thin-walled tube type, thin-walled frame type, etc. The thin-walled cylindrical structure of carbon fiber materials, characterized by lightweight and increasingly mature technology, is widely used in space cameras such as Quickbird-2 [11], ALSAT-2A [12], and KompSat-3 cameras [13]. Generally, the thin-walled frame type is integrally cast or in combination. This structural form often has high structural stiffness and sound structural stability, being generally used for small- and medium-sized off-axis space cameras with complex structural shapes. ALI camera [14] and IKONOS [15] adopt this structural form. The U-shaped frame of the wide-angle auroral imager on the Fengyun 3D satellite adopts CNC machining to achieve a thin-walled cavity structure, and its base support adopts casting methods to achieve a thin-walled frame structure [16]. With the development of space cameras, there is a growing demand for weight reductions. Therefore, it is necessary to design its supporting structural form. The traditional casting and CNC machining methods for thin-walled structures exhibit the disadvantages of long production process cycles and low material utilization, making it difficult to achieve the integrated manufacturing of large and complex thin-walled structures [17,18]. The carbon fiber material lamination process is commonly used to manufacture simple geometric components and will reduce the inherent advantages of carbon fiber composite materials when applied to more complex plate-like structures [19,20].

AM is increasingly widely used in aerospace due to its ability to create complex and customized designs, reduce component weight, improve manufacturing efficiency, shorten product development cycles, and make supply chains more flexible [21,22]. Research into AM in aerospace has also seen exponential growth over the last decade [23]. The aerospace additive manufacturing market industry is projected to grow from USD 8.29 billion in 2023 to USD 36.25 billion by 2032, exhibiting a compound annual growth rate of 20.24% during the forecast period [24]. Ishfaq K. et al. discussed the application of AM in the space field, and these applications demonstrate the feasibility and benefits of AM technology application in space environments [25]. Ransikarcum K et al. conducted a study on the business scope and supply chain scope of AM [26,27]. Wang D et al. investigated the influence of process parameters on densification, microstructure, and mechanical properties of a Ti-6Al-4V alloy printed by SLM [28]. Zheng Li et al. conducted a literature review on the in situ detection, generation, effects, and countermeasures of spatter in L-PBF [29]. Among numerous metal 3D printing technologies, the selective laser melting (SLM) method is very regarded due to its advantages in dimensional accuracy, surface quality, and component performance [30,31].

The efficiency and flexibility of additive manufacturing technology are particularly suitable for space camera structures that require precision and high performance. RUAG designed and produced an optimized version of an existing antenna bracket with AM solutions for the latest editions of the mission, namely, the Sentinel-1C and Sentinel-1D [32]. The space detector SVOM-MXT feature camera contains an enclosed aluminum shield supported by three small support feet produced via additive manufacturing [33]. NASA’s Perseverance Rover carried 11 components fabricated using metal AM techniques. Of these 11 components, 6 are AM-built heat exchangers used in the MOXIE. The 3D-printed parts of the PIXL include its front and back cover, mounting frame, X-ray bench, and bench support, which are hollow, thin-wall structures. The wall thickness of these parts is between 1 mm and 1.1 mm [34]. MENG Hongtao proposes a complex outer baffle space camera based on AM [35]. Silicon carbide is used to develop a space remote-sensing camera via 3D printing technology. The outer envelope of the camera is 430 mm × 370 mm × 450 mm, with a weight of only 13.5 kg [36]. Design and manufacturing methods that integrate optimization with additive manufacturing technology are used to meet the design requirements of being lightweight and displaying high surface-shape accuracy with space mirrors [37,38,39].

Via our review of the above literature, we found that thin-walled structures, being among the primary support methods for space cameras, are currently produced mainly through traditional manufacturing methods such as CNC machining, casting, and composite material weaving. At present, the application of AM technology in space cameras is mainly concentrated on smaller or lower-strength structural components such as brackets, light shields, aperture plates, camera shells, reflectors, etc. Meanwhile, there is relatively little research and application of AM technology on complex thin-walled main load-bearing structural components with high stiffness and large dimensions. Gaps in the existing research are highlighted in Table 1.

This study not only provides a novel perspective for the design and manufacturing of thin-walled structures in space cameras but also expands the application scope of additive manufacturing technology in the aerospace field, providing a practical application of AM technology in the disciplinary area centering thin-walled main supports. The main innovations of this article are as follows:

- For the first time, the laser additive manufacturing of a large-size complex thin-walled structure is applied to the main bearing structure of the space camera.
- Shape optimization is accomplished by optimizing the lateral slope angle of the main support according to the Timoshenko cantilever beam theory.
- An active fitting optimization algorithm is proposed for use to refine the wall thickness of the thin-walled structure. The optimization algorithm not only reduces the number of iterations but also obtains more precise solutions.
- The utilization of additive manufacturing for large-scale, complex, thin-walled structures introduces a novel perspective in both the field of space camera support and AM.

## 3. Design Requirements and Structural Form of the Camera

### 3.1. Key Technical Indexes

The main technical specifications of the wide-field auroral imager (WFAI) are delineated in Table 2.

### 3.2. Camera Structure Form

**Figure 1:**
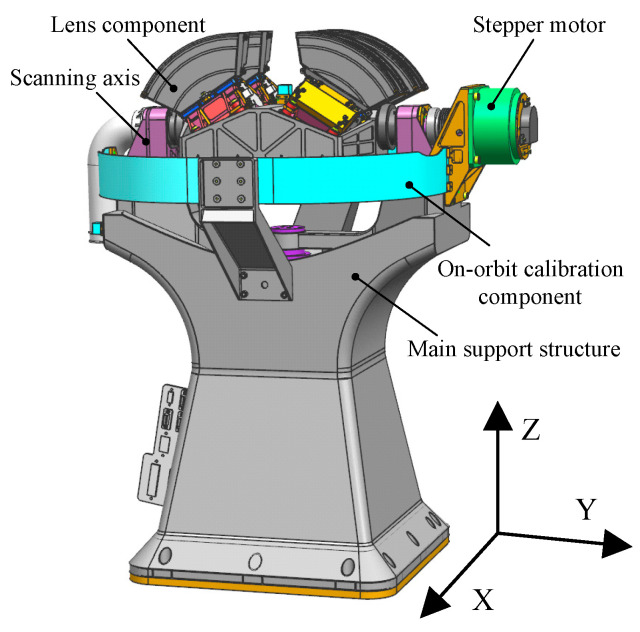
Sketch of the camera structure.

The main constituents of the WFAI are depicted in Figure 1. These components primarily encompass the main support structure, four independent lens components, the scanning axis system, the stepper motor, and the on-orbit calibration component.

The support base plays a pivotal role in the wide-field auroral imager, serving as the primary fixture for various camera components, either directly or indirectly. Notably, the scanning axis system is affixed atop the main support, spanning two relatively independent planes. Additionally, the assembly of four independent lenses, forming the imaging system, is conducted, and the product is positioned within the midsection of the bracket. A motor is responsible for propelling the scanning axis system along the designated track, thereby facilitating the scanning imaging process. Hence, the stability of the main support determines the precision of the shaft, governing the imaging quality of the imaging system and influencing the operational longevity of the camera. Furthermore, in adherence to the specifications outlined in Table 2, the mass of the main support constitutes a substantial 30% portion of the entire camera’s weight. This component also functions as the principal structural element that dictates the camera’s rigidity, considering that other constituents have already undergone extensive lightweight design processes. The primary support structure further benefits from a versatile array of material options and structural configurations within the optical system, rendering it better suited for achieving a high degree of lightweight design when compared to the remaining components.

## 4. Main Support Structure Design

### 4.1. Material Selection

The selection of materials for space cameras primarily revolves around key properties, including excellent structural stiffness and strength, as well as thermal stability [38]. Table 3 illustrates the principal performance indicators of materials frequently employed in space cameras.

It can be seen from the parameters in the table that the specific stiffness of titanium alloy, aluminum alloy, and alloy steel is similar, that the coefficient of linear expansion of aluminum alloy is excessively large, and that the specific strength of alloy steel is small. Although the Invar exhibits superior thermal stability, its specific stiffness and strength are lower. Analyzing the above factors together, it is apparent that the overall performance of titanium alloy is the best. As such, titanium alloy is chosen as the laser additive manufacturing material.

### 4.2. Integrated Design

**Figure 2:**
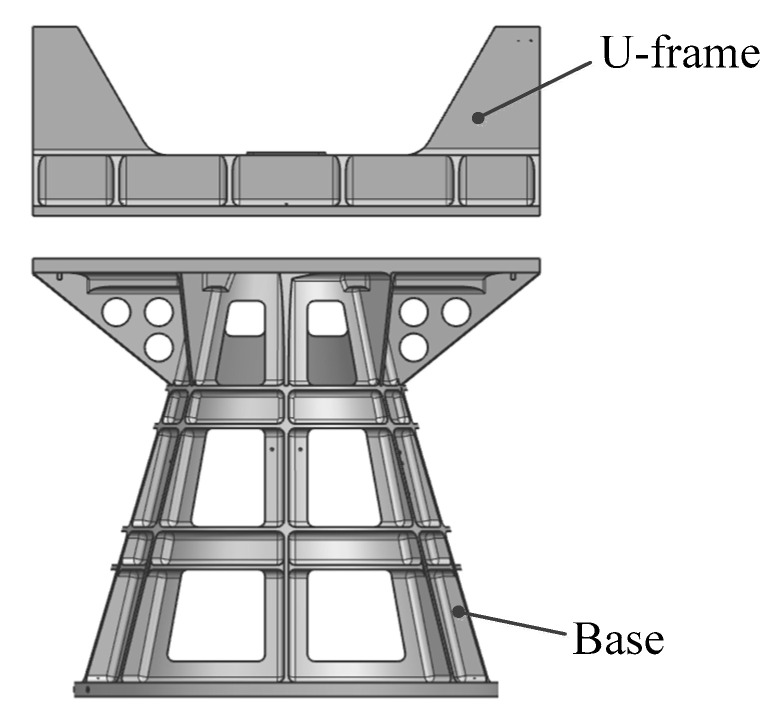
Traditional structural form of main support.

The traditional one-dimensional rotational main support structure comprises two primary components, namely, the U-frame and the base, as depicted in Figure 2. The U-frame serves as the mounting interface for the rotational axis system, while the base acts as the connecting element between the space camera and the satellite platform. The bolted connections used in assembly can compromise the stiffness near the connections, thereby affecting the overall structural dynamics. In terms of manufacturing, in order to achieve lightweight, compact, and intricate structures, CNC machining is commonly employed [41], resulting in material waste and extended processing times. Casting is traditionally used for larger, more complex structures [42], but it often leads to defects, reduced strength, and increased waste rates.

### 4.3. Structural Form Design

**Figure 3:**
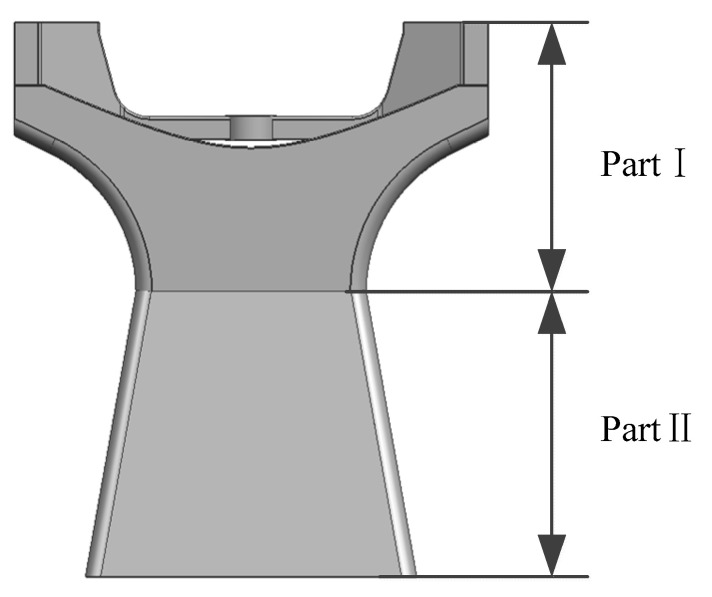
Preliminary structural sketch.

The WFAI achieves a substantial field of view using a swing-sweep mechanism. Consequently, it possesses significant dimensions in the Z-direction, resulting in a relatively high-payload center of mass. Additionally, due to the eccentric load on the scanning mechanism, the main support is subjected to bending and torsional loads, primarily during launch. On occasions when the cross-sectional area is the same, the bending section modulus and torsional section modulus of a thin-walled hollow structure are greater than those of a solid structure. In order to meet the requirements for mass, stiffness, and stability in space cameras, the main support employs a thin-walled box-type structure. Thin-walled box structures are lightweight, offering excellent bending and torsional performances. Internally, these boxes house electronic components, shielding them from radiation, while the exterior surface of the thin-walled box structure provides effective thermal control. The preliminary structural diagram is depicted in Figure 3.

As shown in Figure 3, considering the satellite space size requirements, rotation profile, and optical requirements for on-orbit calibration, the changeable area of Part Ⅰ of the thin-walled beam is small. As such, the optimizable section is Part II. For this reason, this section is designed as a trapezoidal structure. While the overall mass and total height remain unchanged, Part Ⅱ sees evolution from an equal-section rectangle into a variable-section rectangle.

## 5. Optimization

Once the structural form of the main support has been determined, its shape and size should be optimized to achieve the goals of the highest stiffness and lightest mass.

### 5.1. Shape Optimization

#### 5.1.1. Optimization Objective and Variable

Based on the structural design results in shown Section 4.3, the thin-walled main support structure is simplified into a variable cross-section Timoshenko cantilever beam structure. The differential equation for the free vibration of the variable cross-section Timoshenko beam is as follows [43]:

(1) $$\left\{\begin{array}{c} \frac{\partial }{\partial x}\left[\chi GA\left(x\right)\left[\varphi \left(x,t\right)-\frac{\partial y\left(x,t\right)}{\partial x}\right]\right]+\rho A\left(x\right)\frac{\partial^{2}y\left(x,t\right)}{\partial t^{2}}=0\\ \frac{\partial }{\partial x}\left[EI\left(x\right)\frac{\partial \varphi \left(x,t\right)}{\partial x}\right]+\chi GA\left(x\right)\left[\frac{\partial \varphi \left(x,t\right)}{\partial x}-\varphi \left(x,t\right)\right]-\rho I\left(x\right)\frac{\partial^{2}\varphi \left(x,t\right)}{\partial t^{2}}=0 \end{array}\right.$$

This is the coupled equation of motion for a Timoshenko beam, where A(x) is the cross-sectional area, E is the modulus of elasticity, G is the shear modulus, χ is a coefficient related to the shape of the cross-section, ρ is the density of the beam, I(x) is the moment of inertia of the cross-section, y(x,t) is the displacement of the beam, and φ(x,t) is the angle of rotation of the beam cross-section.

Adopt the separated variable approach and bring the following equation into Equation (1).

(2) $$\left\{\begin{array}{c} y\left(x,t\right)=\bar{Y}(x)sin\;(\bar{\omega }t+\gamma )\\ \varphi \left(x,t\right)=\bar{\Phi }(x)sin\;(\bar{\omega }t+\gamma ) \end{array}\right.$$

Then, derive the second part of Equation (1) for x. The principal mode function of the vibration of the beam can be expressed as:

(3) $$\left\{\begin{array}{c} -\frac{\partial }{\partial x}\left[\chi GA\left(x\right)\left(\bar{\Phi }-\bar{Y}\prime \right)\right]+\bar{\lambda }\rho A\left(x\right)\bar{Y}=0\\ \frac{\partial^{2}}{\partial x^{2}}\left(EI\left(x\right)\bar{\Phi }\prime \right)-\frac{\partial }{\partial x}\left[\chi GA\left(x\right)\left(\bar{\Phi }-\bar{Y}\prime \right)\right]+\bar{\lambda }\frac{\partial }{\partial x}\left(\rho I\left(x\right)\bar{\Phi }\right)=0 \end{array}\right.$$

where the eigenvalue $\bar{\lambda }=\omega^{2}$ for a variable cross-section Timoshenko beam. Thus, the principal factors affecting the natural frequency are I(x) and A(x). For an arbitrary-variable cross-section beam, solving the analytical solution of Equation (3) is usually difficult because Equation (3) is a system of differential equations with variable coefficients.

**Figure 4:**
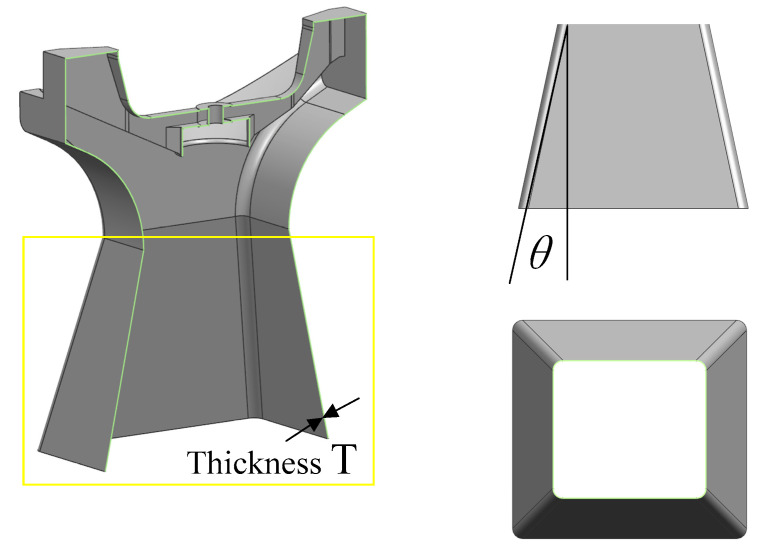
Main support optimization variables.

The objective underlying shape optimization is to obtain a first-order natural frequency as high as possible. From the above equation and Figure 4, it can be seen that Part Ⅰ of the thin-walled beam possesses a small changeable area, meaning that the optimizable section is Part II, which are in the yellow square. In Part II, any cross-section shape is a thin-walled rectangular cross-section, and as the angle θ between the side of the main support and the vertical plane (lateral slope angle) changes, the thin-walled rectangular cross-section dimensions evolve also. Therefore, the cross-sectional moment of inertia also exhibits alterations. For this reason, the lateral slope angle θ is the optimization variable of shape optimization.

#### 5.1.2. Optimization Strategy

The optimization of lateral slope angle θ is conducted with the objective of obtaining the highest first-order natural frequency of the support base for a given base mass and height. In this paper, the optimization strategy is an ergodic method. This involves determining the range of values of θ and then choosing the ergodic step size Δθ. Then, it is necessary to calculate the different wall thickness Ti corresponding to different θi with the given mass Md of the support base. Next, researchers should derive the first-order natural frequency ωi corresponding to θi according to the free vibration equation of the support base. Finally, the relationship between the lateral slope angle θ and the first-order natural frequency ωi is to be obtained by traversing all the values of θ, thus determining the optimum lateral slope angle θ and the first-order natural frequency ωi.

#### 5.1.3. Optimization Process and Result

**Figure 5:**
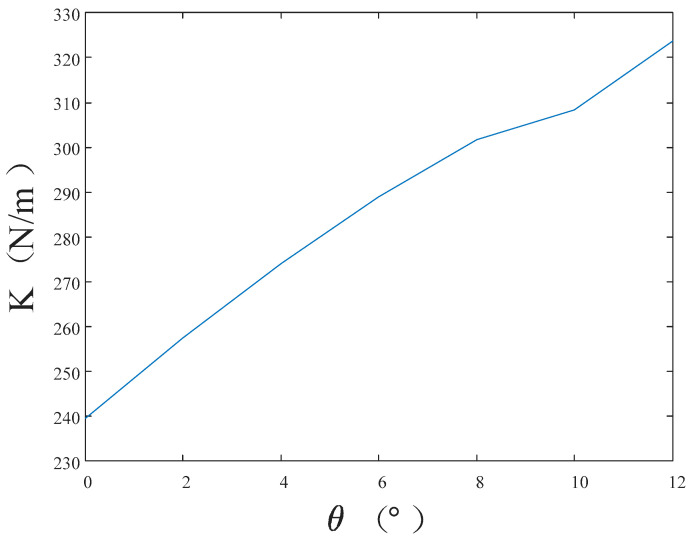
Optimization results of the lateral slope angle.

The optimization process of the lateral slope angle θ and the results thereof are as follows. Initially, the permissible range for θ, based on the satellite installation space, lies between 0° and 12°. Then, a step size of 2° is selected for the exploration. The mass of Part Ⅱ of the main support structure is set at 4 kg. The corresponding wall thickness T can be calculated from the total mass and θ. Subsequently, for each θ value within the specified range, the corresponding free vibration equation for Part Ⅱ of the main support structure is formulated. By solving this equation, the first-order natural frequency for each θ value is obtained. The optimization results are illustrated in Figure 5. As observed in the graph, the optimal lateral slope angle (θ) is determined to be 12°, with a corresponding wall thickness of 1.779 mm.

### 5.2. Structural Reinforcement Design

**Figure 6:**
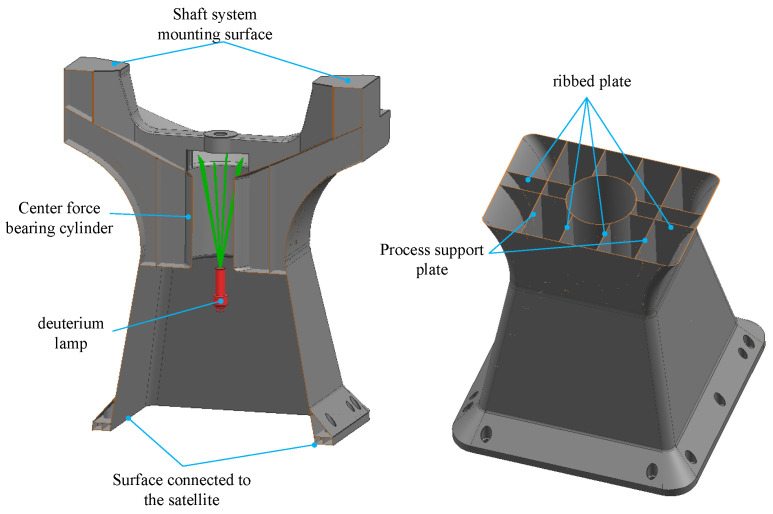
Structural reinforcement of the main support.

The primary photomechanical structure of the space camera is arranged on Part Ⅰ of the main support, and its strength, stiffness, and stability must overcome the vibration of the mechanical environment of the rocket launching stage. Otherwise, the accuracy of the shaft system cannot be guaranteed, which in turn affects the imaging quality of the optical system and the working life of the camera. Therefore, the cavity of Part Ⅰ is structurally strengthened, as shown in Figure 6. The inside cavity of Part Ⅰ mainly consists of the center force-bearing cylinder, eight ribs, etc., which together form the main force transmission path. The scanning axis system, stepping motor, and calibration components transfer the load to the main force transmission path. Meanwhile, for the purpose of preventing stress concentration, the shaft mounting surface and satellite connection surface are thickened to 3 mm, and other wall thicknesses are designed according to the optimization result of 1.779 mm. At this time, the mass of the main support is 10,615 Kg. In order to conduct additive manufacturing, an auxiliary support plate is designed, and a rounded design is performed in the thin-wall connection to prevent cracking during laser printing. Simultaneously, the center bearing cylinder also plays the role of supporting the calibration of the deuterium lamp assembly and the role of the shading cylinder.

### 5.3. Size Optimization

#### 5.3.1. Optimization Objectives and Variables

Shape optimization ensures the high stiffness of the main support, while size optimization accomplishes the goal of lowering the weight of the support base.

The objective of size optimization is to minimize the mass of the main support base. Simultaneously, for the purpose of avoiding resonance phenomena during the rocket’s launch that could potentially damage the structure, the first-order natural frequency of the camera is required to be no less than 120 Hz. Therefore, the constraint for the optimization problem is that the first-order natural frequency of the main support base must exceed 120 Hz. The wall thickness of thin-walled structures is the most important factor affecting the mass of the main support. As a consequence, the wall thickness T is used as an optimization variable for dimensional optimization.

#### 5.3.2. Optimization Strategy

The wall thickness T of the support base is used as the optimization variable, the minimum mass is used as the optimization objective, and the first-order natural frequency of the main support is kept higher than 120 Hz as a constraint for optimization.

The conventional dimensional optimization method involves giving an initial wall thickness T_0_ and then increasing the thickness ∆t each time until the resulting thickness meets the set requirement. The next step involves outputting the wall thickness at a minimum volume, denoted as follows:

(4) $$s.t\left\{\begin{array}{c} Mass_{min}(T_{0}+∆t\times N)\\ f_{1}(T_{0}+∆t\times N)\geq 120\;Hz \end{array}\right.$$

where T_0_ is the initial wall thickness, ∆t is the wall thickness increment, and N is the number of iterations.

However, there remain some problems with this approach. If ∆t takes a large value, although the number of iterations can be reduced, the solution may be rough; conversely, if ∆t takes a small value, although a detailed solution can be obtained, the number of iterations will be increased, consuming a lot of computational resources.

For this reason, we propose an active fitting optimization algorithm, i.e., the active fitting of the polynomial function is performed after each calculation, the wall thickness at the target frequency is solved numerically, and the minimum wall thickness is selected for calculation. These steps not only reduce the number of iterations but also achieve a detailed solution. The theory of the active fitting optimization algorithm is illustrated in Algorithm 1.
**Algorithm 1:** Active Fitting Optimization AlgorithmInput: T is the wall thickness of the model, FRE is the 1st-order mode of the model, N is the maximum number of iterations, M is a polynomial number, ${FRE}_{target}$ is the target value of the 1st-order mode of the model, $R_{target}^{2}$ is the fit threshold, and ∆FRE is the residual value. Output: T. 1: $T_{0}=\emptyset ,T_{1}{=\emptyset ,T}_{1}\neq T_{0}$; calculations using the finite element method ${FRE}_{0}(T_{0}),{FRE}_{1}(T_{1})$//Step 1. Calculation of 1st-order modes ${FRE}_{0}$ and ${FRE}_{1}$ for $T_{0}$ and $T_{1}$ wall thicknesses using finite element methods 2: N=∅; $M=\emptyset ;{FRE}_{target}=\emptyset$;//Step 2. The variable assignment cannot be 0. 3: for (i = 1 to N) do 4: From [$T_{0}{FRE}_{0}$]∼[$T_{i}{FRE}_{i}$], calculate the ${F_{i}(t)=p}_{M}t^{M}+\cdots +p_{2}t^{2}+p_{1}t+p_{0}=\sum_{j=0}^{M}p_{j}t^{j}$; //Step 3. Constructing the fitting function 5: Calculate the degree of fit $R^{2}$;//Step 4. Calculating goodness of fit 6: if ${(R}^{2}\geq R_{target}^{2}$) then 7:  when $F_{i}(t)={FRE}_{target}$ solve ($t_{0}t_{1}{\cdots t}_{n}$); 8: $T_{i+1}^{min}$ = ($min(t_{0}t_{1}{\cdots t}_{n}$)∪t>0); 9: Set the wall thickness of the model as $T_{i+1}$, and use the finite element method to calculate the 1st-order mode as ${FRE}_{i+1}$; 10:  if ${|FRE}_{i+1}-{FRE}_{target}|\leq ∆FRE$) then 11: end for; else 12:   [$T_{i+1}{FRE}_{i+1}$] = [$T_{i+1}^{min}{FRE}_{i+1}$]; 13:  end if 14: else 15:  i=i+1; 16:  M=M+1; 17: end if 18: end for 19: return $T_{i+1}$, i, N

#### 5.3.3. Optimization Process and Result

**Figure 7:**
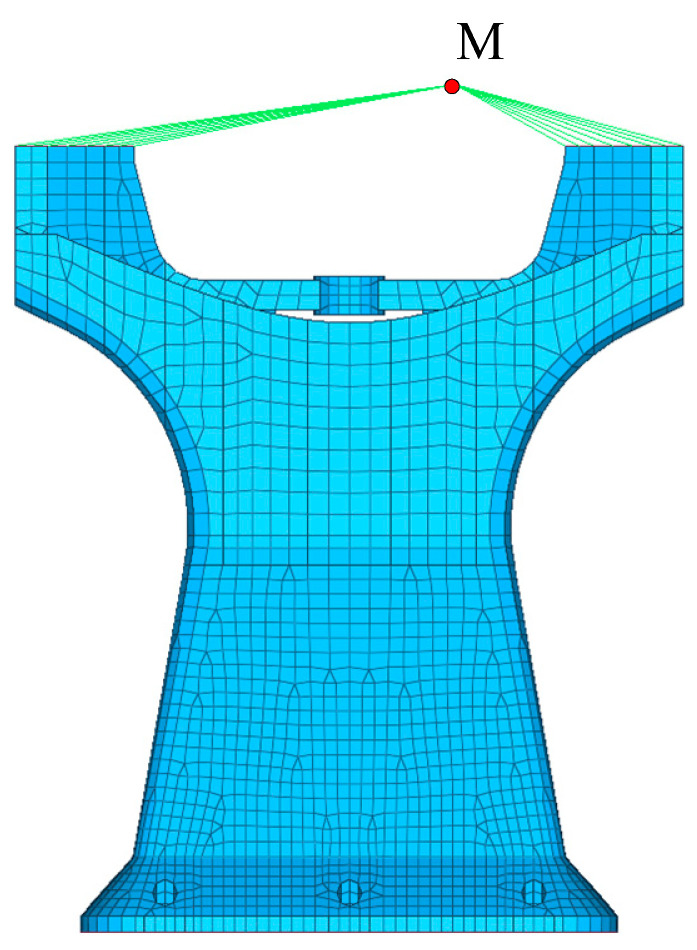
Finite element model.

The optimization process and results of the wall thickness T are as follows. The principal support structure was divided into shell elements. For the sake of simplification, all structural components at the cantilever end were condensed into a single mass node known as M. This approach took into consideration the eccentricity of the mass node and incorporated moments of inertia in three directions. Care was taken to maintain precise connection arrangements, resulting in the simplified finite element model depicted in Figure 7.

**Figure 8:**
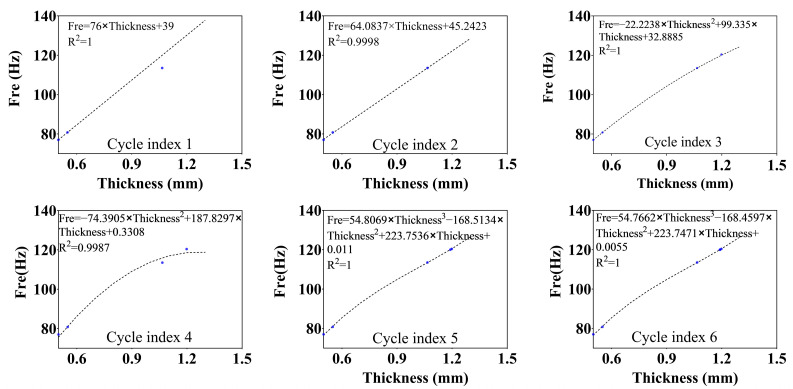
Changes in the fitted curves during the optimization process.

**Figure 9:**
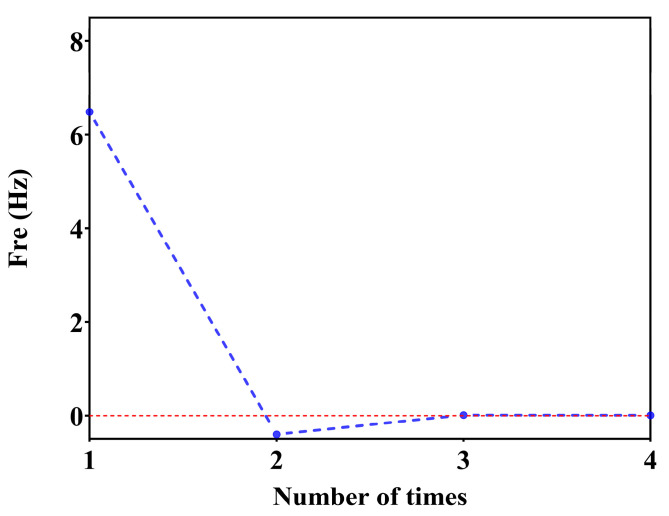
Frequency residuals in the convergence process.

The active fitting optimization algorithm is used to calculate the optimization of the model wall thickness. Firstly, the 1st-order mode for wall thickness T_0_ = 0.5, T_1_ = 0.55 is calculated as FRE_0_ (T_0_) = 77, FRE_1_ (T_1_) = 80.8, the number of cycles is set to be N = 10, the number of polynomials is set to be M = 1, the target value for the 1st-order mode of the model is set to be ${FRE}_{target}=$120, the goodness of fit is set to be $R_{target}^{2}=0.9999$, and ∆FRE = 0.01 is the residual value. After six cycles and four finite element calculations, the 1st-order mode FRE = 119.9927 Hz of the model is obtained for a wall thickness of T = 1.19 mm. The variation of the fitting curve is shown in Figure 8, and the frequency residual curve during convergence is shown in Figure 9.

### 5.4. Mechanical Analysis

The structural design of the support base was completed based on the optimized wall thickness obtained in the previous section. The total mass of the main support structure was 7.56 kg, resulting in a 28% reduction in camera mass. Such a measure ensures that the structure’s natural frequency remains within the optimization constraints while also helping to significantly reduce overall camera weight. Following the optimization, local reinforcement design was applied to the camera’s mounting locations, and stress concentration design modifications were conducted at threaded connection points.

**Figure 10:**
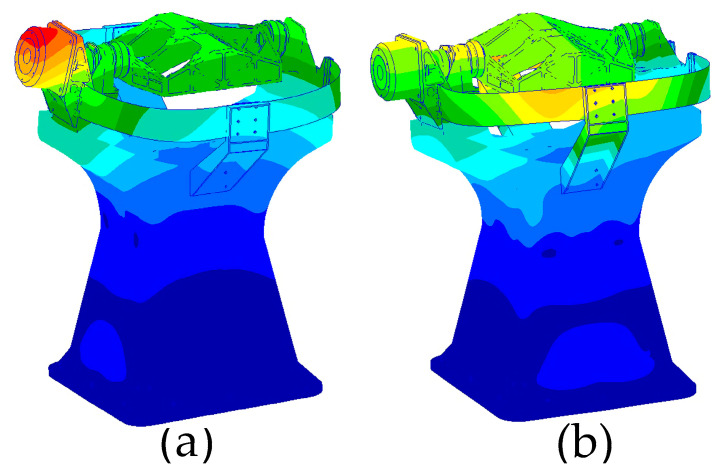
Horizontal modal vibration pattern of space camera. (**a**) Y direction natural frequency, (**b**) X direction natural frequency.

In the optimization design, the load was considered as a point mass, and the rotational inertia of the load was not taken into account. However, in the actual system, there is some flexibility in the load, shaft system, and their connections. As a result, the natural frequencies of the actual system are lower than the optimized results. To obtain a more accurate representation of the system’s natural frequencies, it was necessary to conduct a modal analysis of the entire system. The 11 connection holes at the bottom of the main support were constrained, and the system’s natural frequencies in two horizontal directions were calculated to be 109.2 Hz and 110.5 Hz, as illustrated in Figure 10. The results indicate that the first-order natural frequency, calculated using a finite element model with realistic loads, is 10.8 Hz lower than the results obtained via consideration of point masses.

## 6. Laser Additive Manufacturing and Experimental Verification

### 6.1. Laser Additive Manufacturing

Compared with the traditional one-dimensional scanning main support structure, which consists of a U-frame and a base, the optimized main support structure adopts integrated thin-wall structures, such as the thin-wall structures shown in Figure 4 and Figure 6, whose overall size reaches 538 mm × 400 mm × 384 mm. The wall thickness is 1.19 mm, and there are some closed cavity structures. It is almost impossible to complete the work using conventional processing means. Additive manufacturing can overcome the problems of chattering in traditional thin-wall CNC machining and quality defects in thin-wall casting. As such, this paper chooses selective laser melting technology to manufacture this structure.

**Figure 11:**
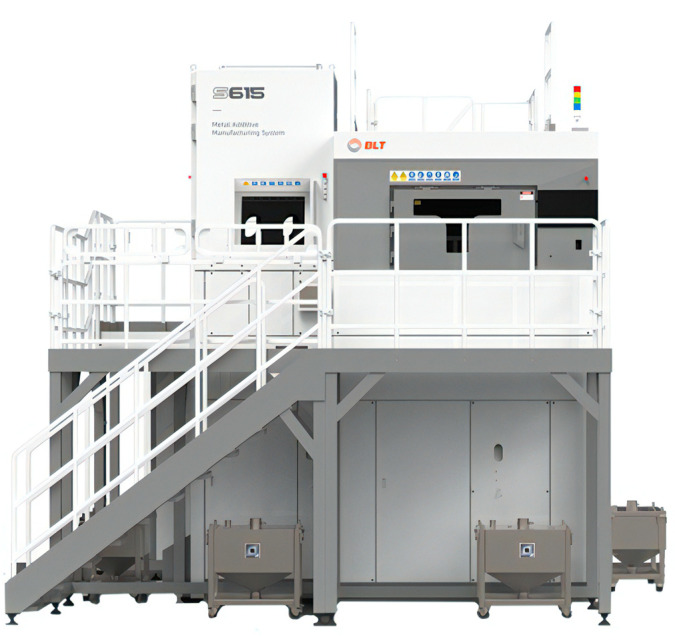
The BLT-S615 multi-metal 3D printing system.

Given that the main support structure was constructed from titanium alloy Ti6Al4V material and possesses substantial dimensions, with an outer envelope measuring 538 mm × 400 mm × 384 mm, a comprehensive assessment of the available large-scale 3D printing equipment was conducted. After thorough consideration, the decision was made to employ the BLT-S615 multi-metal 3D printing system manufactured by Bright Laser Technologies (Xi’an, China). The 3D model of this 3D printing device is shown in Figure 11, the specific parameters of which are outlined in Table 4.

BLT-S615 equipment possesses a maximum size of 600 mm × 600 mm × 1500 mm, meeting the required dimensions for the main support structure. It features a laser power of 500 W × 4, a laser wavelength in the range of 1060 nm to 1080 nm, and a maximum scanning speed of 7 m/s. Additionally, achievable part accuracy can be controlled within ± 0.05 mm. Therefore, the BLT-S615 was selected for the manufacturing of the main support structure using metal additive manufacturing technology. The quality met design specifications. The specific experimental print parameters of the thin-walled main support structure by SLM in this paper are shown in Table 5. After the completion of printing, the ultrasonic thickness gauge is utilized to measure part of the wall thickness. The thickness range is from 1.21 mm to 1.25 mm, and the error is within the allowable range. The mass of the main support structure is 7.78 Kg, which is slightly greater than the design weight but still meets the design requirements.

### 6.2. Experimental Verification

**Figure 12:**
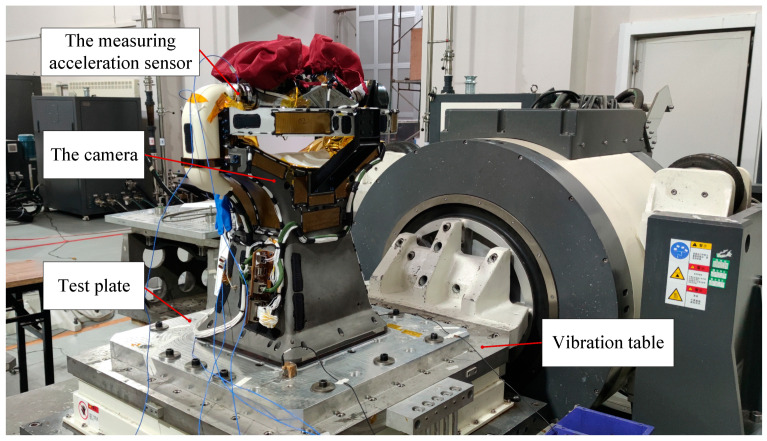
Sweep frequency vibration test site.

**Figure 13:**
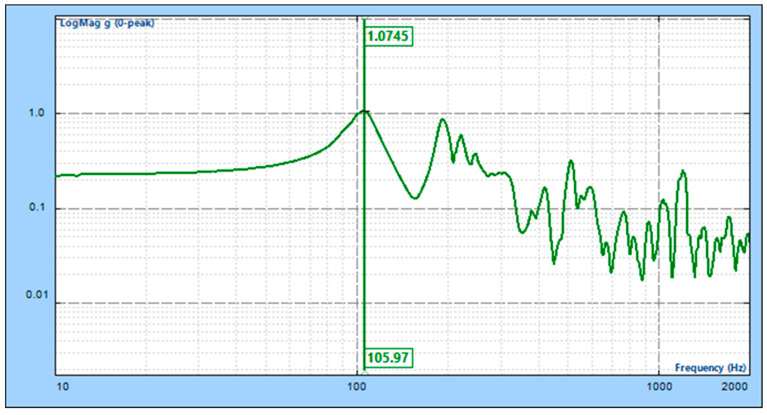
X-direction sweep test results.

To test the system’s inherent frequency in the horizontal direction, a horizontal 0.2 g sweep frequency vibration test was conducted using an electromagnetic vibration table, as depicted in Figure 12. The results of the camera’s horizontal sweep frequency test are shown in Figure 13. The camera’s inherent frequencies in the horizontal direction were determined to be 105.97 Hz, with an error of 3% compared to the simulated result of 109.2 Hz, validating the optimized design results presented in this paper.

## 7. Conclusions and Future Research

In this paper, we designed the main support structure for a wide-angle auroral imager with an integrated thin-wall structure via structural optimization. We then manufactured this via selective laser melting (SLM). First, this marked the first application of laser additive manufacturing of large-size complex thin-walled structures to the production of the main bearing structure of a space camera. The processed size of the main support structure reached 538 mm × 400 mm × 384 mm, and the wall thickness of the thin-walled structure was 1.19 mm. The vibration test proved that the structure exhibited good stiffness with lighter mass. Second, the initial design of the thin-walled main support was simplified to have a Timoshenko cantilever beam structure. The study analyzed the principal parameters affecting the natural frequency according to Timoshenko beam theory, subsequently optimizing the angle between the side of the main support and the vertical plane. Finally, an active fitting optimization algorithm was proposed for the refinement of the thin-walled structure’s wall thickness. The optimization algorithm not only reduced the number of iterations but also obtained more precise solutions. The proposed methodology provides valuable insights and references for the future design and manufacturing of similar space optical camera sensors. Furthermore, the approach of utilizing additive manufacturing to produce large-scale complex thin-walled structures introduces a novel perspective in both the field of space camera support and AM.

This paper presented a case study of a large-scale complex thin-wall main support structure based on additive manufacturing in a space camera. The primary focus of the research lay in the design and optimization of the main support structure. During the research process, several other aspects deserving analysis were identified. Firstly, it was crucial to determine precisely how process parameters used in laser additive manufacturing, such as powder materials, laser power, scanning velocity, hatch spacing, and powder layer thickness, impact both the quality and frequency of the space camera as well as its structural stress. Secondly, because of the thin wall thickness and large size of the main support structure, the thermal stress in the laser AM process can easily cause deformation of the overall structure. Therefore, this article also discusses how to reduce thin-walled deformation by designing and controlling process parameters in laser AM. Lastly, relevant experiments must be conducted to verify their thermal stability and mechanical stability due to thin-walled structures being prone to deformation, buckling, and other problems in environmental tests.

## Figures and Tables

**Table 1:** Existing studies and research gaps in thin-wall structures in space cameras.

Research Topic	Existing Research	Research Gaps
Structural type design	Thin-walled cylinder type [11,12,13]	Integrated complex thin-walled structure designs
	Thin-walled frame type [14,15]	
Traditional manufacturing technology	CNC machining and casting [16]	Low-cost, high-quality, high-efficiency and pollution-free in space
	Composite material weaving [11,12,13]	
Additive manufacturing applications	Small-scale functional components [32,33,35,36,37]	Applications in large-scale, high-rigidity, and thin-wall primary load-bearing structures
	Strength assessment components [30]	
	Small-scale support structure [31,34]	

**Table 2:** Main technical specifications of the camera.

No.	Parameters	Specifications
1	Mass (kg)	≤25
2	Frequency (Hz)	≥100
3	Volume (mm)	≤726 × 635 × 500

**Table 3:** Material parameters of space support structure [40].

Name	Densities ρ(g·cm^−3^)	Modulus *E* (Gpa)	E/ρ (kN·m/g)	Expansivity (10^−6^ K^−1^)	Tensile Strength	Specific Strength
TC4	4.44	110	24.7	8.8	802	180.6
Al alloy	2.71	69	25.5	23	410	146.4
Alloy steel	7.83	210	26.9	12	780	100
Invar	8.1	145	17.9	2.4	302	37.3

**Table 4:** Parameters of the BLT-S615 multi-metal 3D printing system.

Material Support	Titanium Alloy, Aluminum Alloy, High-Temperature Alloy, Stainless Steel, High-Strength Steel, Tool Steel
Forming size	600 mm × 600 mm × 1500 mm (W × D × H), 650 mm × 650 mm × 1300 mm (W × D × H)
Power of the laser	500 W × 4, 500 W × 6
Layering thickness	20 μm∼100 μm
Maximum scan speed	7 m/s
Efficiency of forming	100 cm^3^/h
Preheating temperature	RT + 20∼200 °C
Beam quality	M2 < 1.1
Optical structure	F-θ footage
Powder laying agencies	Single/two-way spreading of powder
Power wastage	≤18 KW
Size	4700 mm × 5100 mm × 3800 mm (W × D × H)
Weight	≈14,900 kg

**Table 5:** Process parameters of the main support structure by SLM.

Device Model	Laser Power	Spot Diameter	Powder Layer Thickness	Scanning Velocity	Hatch Spacing	Powder Materials
BLT-S615	4 × 340 W	80–85 μm	60 μm	1250 mm/s	120 um	Ti6Al4V

## Data Availability

The data presented in this study are publicly available in the article.
